# Navigating the landscape of academic prose: A corpus-driven inquiry into rhetorical preferences and their pedagogical implications for advanced L2 writers

**DOI:** 10.1371/journal.pone.0343739

**Published:** 2026-03-05

**Authors:** Yang Yu, Yingying Xu, Yongkang Wu

**Affiliations:** School of Foreign Languages, Dalian Maritime University, Dalian, China; National University of Malaysia Faculty of Education: Universiti Kebangsaan Malaysia Fakulti Pendidikan, MALAYSIA

## Abstract

This study presents a pedagogically motivated inquiry into the cross-cultural rhetorical patterns of academic writing, focusing on the use of general nouns (GNs). The research was initiated in response to persistent difficulties observed among advanced L2 writers, who struggle to use GNs with appropriate nuance to establish an authoritative stance. Employing a corpus-driven methodology, the study analyzes two purpose-built corpora: the Chinese Academic Written English Corpus (CAWEC) and the Inner-Circle Affiliated Written English Corpus (ICAWEC). The findings reveal divergent rhetorical tendencies. Writers in the CAWEC show a statistically significant preference for “Research-group” (e.g., *study*, *research*) and “Result-group” nouns (e.g., *difference*, *results*). Their collocational patterns, marked by temporality (*current study*) and subjectivity (*our study*), are consistent with a conceptual metaphor of ACADEMIC PROGRESS IS A JOURNEY. In contrast, writers in the ICAWEC use “Example-group” (e.g., *case*, *fact*) and certain “Discussion-group” nouns (e.g., *argument*) more frequently, dominated by objectifying collocations (*the study*). These patterns suggest a spatialized argumentative strategy consistent with a conceptual metaphor of THE STUDY IS A KNOWLEDGE CONTAINER. By making these frameworks explicit, the study proposes a pedagogical model to expand L2 learners’ rhetorical repertoires and metacognitive awareness, equipping them to navigate Anglophone academic discourse.

## 1. Introduction

The increasing globalization of research has firmly established English as the undisputed lingua franca of international academic communication. For tertiary-level students and emerging scholars who are non-native English (NNE) speakers, the development of advanced academic literacy in English represents a critical gateway to participation in the global discourse community. Effective academic communication transcends mere grammatical accuracy; it demands a sophisticated command of the rhetorical conventions, lexico-grammatical nuances, and implicit epistemological stances that characterize a given disciplinary field [1]. As scholarship by Hyland [2] and Swales [3] has extensively documented, the quality of language use directly shapes the reception, credibility, and ultimate impact of research. This reality imposes a significant burden on second language (L2) writing programs worldwide, which are tasked with cultivating these advanced linguistic and rhetorical competencies.

This research project was born directly out of a persistent pedagogical challenge encountered within a third-year undergraduate “Advanced English” course at a major Chinese university, a course populated primarily by advanced L2 writers of English. A primary objective of this course is the development of students’ academic writing capabilities, facilitated through a series of research-based writing assignments. Over successive academic years, a consistent pattern of difficulty emerged concerning the use of general nouns (GNs). These lexical items—such as *study, research, fact, issue, result, process, problem,* and *argument*—are deceptively complex. While they appear to be simple vocabulary, their function is profoundly context-dependent, serving as crucial devices for structuring arguments, signaling authorial stance, and establishing an authoritative writer’s voice [4].

The choice to focus specifically on General Nouns rather than other cohesive devices is deliberate. Unlike transition signals, which function primarily as logical connectors, or modal verbs, which modify the truth value of a proposition, GNs possess a unique dual function: they are simultaneously cohesive devices and carriers of propositional content [5]. The concept of “shell nouns” [5] or “signalling nouns” [6] captures this functional complexity, where the noun acts as a conceptual “shell” whose specific meaning and rhetorical force are determined by its surrounding linguistic environment. They allow writers to encapsulate complex segments of text into a single nominal unit (e.g., *this unexpected result*) and retrospectively label it with an evaluative stance. This capacity makes them a far more subtle and challenging indicator of advanced academic literacy than purely grammatical features.

Our students’ written work frequently demonstrated a constrained repertoire of these GNs, a high incidence of unconventional collocational patterns, and a general failure to harness their full rhetorical power to project a convincing academic persona. For example, a common pattern was the monotonous overuse of subjective, process-oriented phrases like “our research shows that...” or “this study aims to...”, coupled with a less frequent use of the more objectified, authoritative constructions often favored in Anglophone academic prose, such as “the study reveals that...” or “the evidence suggests that...” This specific difficulty is not an isolated anecdote but is symptomatic of a broader challenge well-documented in L2 writing research: learners often struggle to move beyond surface-level grammatical and lexical knowledge to master the idiomatic, phraseological, and rhetorical subtleties essential for successful academic socialization [7].

Consequently, the primary purpose of this study is to transcend a superficial cataloging of student difficulties and instead explore the systematic, underlying rhetorical patterns that may inform them. This study hypothesizes that the observed patterns in L2 writing may be partly informed by rhetorical conventions prevalent in L1 expert academic writing, and that a systematic comparison can reveal points of divergence that are pedagogically valuable for raising students’ rhetorical awareness. To explore this hypothesis, the study employs a corpus-driven, comparative methodology, analyzing the professional academic writing of expert Chinese scholars (serving as a proxy for L1-influenced patterns) and expert scholars affiliated with inner-circle English institutions (serving as a baseline for high-frequency conventions in the target discourse).

The rationale for comparing two expert groups is not to establish a “native” standard as a superior benchmark, but rather to use the Anglophone-affiliated expert corpus as a data-driven baseline to identify common conventions in the target discourse communities these L2 writers aspire to join. The Chinese expert corpus is used to explore whether some of the patterns observed in student writing might reflect established rhetorical traditions rather than being random errors. This approach treats the investigation as an exploratory, diagnostic step for curriculum design, not a deficit analysis of L2 writing.

The significance of this research is therefore primarily pedagogical, with direct implications for the L2 writing classroom. Theoretically, it aims to contribute to the intersecting fields of EAP, cognitive linguistics, and second language writing by illustrating how abstract rhetorical preferences—which can be interpreted through either temporal or spatial framing of knowledge—are systematically realized in concrete lexico-grammatical choices. By framing the analysis around two master conceptual metaphors, ACADEMIC PROGRESS IS A JOURNEY and THE STUDY IS A KNOWLEDGE CONTAINER, the research offers a robust heuristic model for understanding cross-cultural rhetorical variation. This approach elevates the discussion from simple notions of L1 transfer to a more sophisticated exploration of how language, culture, and cognition may interact to shape the very construction of knowledge itself, echoing the broader inquiries of cultural cognition researchers like Nisbett [8].

Practically, and most crucially, the findings of this study are intended to serve as a direct, evidence-based foundation for pedagogical reform within the “Advanced English” course and similar L2 writing programs. By pinpointing specific, high-frequency points of divergence in GN usage, the research can design and implement targeted instructional materials and classroom interventions. The ultimate pedagogical objective is to cultivate in students a “metaphorical awareness” [9], enabling them to perceive that mastering academic English necessitates not only the acquisition of new vocabulary and grammatical structures but also an understanding of, and the ability to flexibly navigate, the different cognitive and rhetorical pathways that writers use to build and present arguments in different cultural contexts. This study, therefore, functions as a foundational needs analysis for a more cognitively-informed and rhetorically-aware approach to L2 writing pedagogy.

To fulfill these research and pedagogical aims, the study is structured around four guiding questions:

(1) What are the statistically significant differences in the frequency and distribution of semantic groups of general nouns used in academic articles by Chinese scholars versus scholars affiliated with inner-circle English institutions?
(2) What are the distinct collocational patterns (specifically L1 collocations) associated with these general nouns in each corpus, and what do these patterns reveal about their preferred local discourse functions?
(3) To what extent can the observed differences be interpreted through the lens of conceptual metaphor theory, and what alternative explanations, such as L1 rhetorical and grammatical transfer, might also account for these patterns?
(4) Based on these findings, what specific pedagogical interventions can be designed to enhance L2 learners’ awareness and control of these nuanced patterns in their own academic writing?

## 2. Theoretical framework

### 2.1 General nouns in academic discourse

General nouns (GNs) constitute a distinct and functionally versatile lexical category whose precise semantic interpretation is exceptionally dependent on the co-text in which they appear. Halliday and Hasan [10], in their seminal work on cohesion, were among the first to formally identify GNs, defining them as a small class of nouns with broad denotative meaning that serve a critical generalizing function in discourse. Their primary significance lies in their capacity for “discourse referentiality”—the ability to anaphorically encapsulate and refer back to extensive segments of preceding text. A single noun like *issue, phenomenon,* or *finding* can thus package complex propositions into a manageable nominal unit, contributing immensely to the cohesive flow and thematic development of a text.

Subsequent linguistic inquiry has elaborated on this core function from multiple theoretical perspectives. Working within a discourse analysis framework, Francis [11] further investigated their anaphoric role, coining the term “anaphoric nouns” and metaphorically describing them as textual “signposts” that guide the reader’s attention. Expanding on this, Ivanič [12] introduced the concept of “carrier nouns,” a term that emphasizes their semantic flexibility; these nouns “carry” specific meanings derived entirely from their immediate linguistic context. This line of research was significantly advanced by Francis’s [13] later work on “retrospective labelling” and “prospective labelling,” which provided a more systematic account of how these nominal devices function to organize textual information.

A highly influential development in this area has been the conceptualization of GNs as “shell nouns” [5] or “signalling nouns” [6,14]. Schmid [5], through extensive analysis of large-scale corpora, proposed that these nouns function as conceptual “shells” that are “filled” with specific semantic content by their surrounding linguistic material (e.g., a *that*-clause or a prepositional phrase). This model provides a powerful cognitive framework for understanding their context-dependency. Building on this, Flowerdew’s extensive work [6,15,16] has meticulously cataloged the forms and functions of “signalling nouns” in academic discourse, establishing a detailed classification system. This body of research has been instrumental in revealing the crucial role GNs play in academic argumentation, not just as cohesive devices but as tools for constructing an authorial stance, evaluating information, and managing reader expectations. For L2 writers, the challenge is immense, as the effective use of these nouns is often subtle, idiomatic, and deeply enmeshed in the tacit rhetorical conventions of a specific discourse community [4,17].

### 2.2 Collocation and local grammar

Understanding the functional behavior of GNs is impossible without considering their habitual lexical partnerships. The meaning and rhetorical function of a GN are rarely inherent in the noun itself but are activated and shaped by the words with which it regularly co-occurs. The study of collocation—defined by Firth [18] as the frequent and predictable co-occurrence of lexical items—is therefore indispensable. Sinclair’s [19] “idiom principle” posits that a significant portion of language production relies not on generating utterances word-by-word from abstract grammatical rules, but on deploying pre-constructed or semi-preconstructed phrases. For GNs, their collocates are what specify their function in any given instance. For example, the GN *study* is functionally transformed by its L1 collocate: *the study* objectifies it, *our study* personalizes it, *this study* points to its immediate textual presence, and *a previous study* positions it historically.

To systematically capture these specific, function-driven linguistic patterns, this study adopts the methodological framework of “local grammar” [19,20]. Unlike traditional grammars that aim to formulate universal rules, a local grammar is a descriptive model tailored to a specific semantic or pragmatic domain. It focuses intensely on the particular lexico-grammatical resources used to realize a particular discourse function. By meticulously analyzing the recurring collocational patterns surrounding a set of functionally related words (like GNs), one can reveal systematic patterns of use that would remain invisible to a more generalized grammatical analysis [21]. This approach is intrinsically corpus-driven, allowing the functionally significant patterns to emerge organically from authentic language data rather than being imposed by pre-existing theoretical categories.

### 2.3 Cognitive linguistics and conceptual metaphor

While local grammar provides a powerful methodology for describing what the linguistic patterns are, cognitive linguistics offers a robust theoretical framework for explaining why they might exist. A foundational principle of cognitive linguistics is that language is not an autonomous module separate from other cognitive functions; rather, it is a direct reflection of our overall conceptual system, which is itself grounded in our embodied experiences and shaped by our cultural context [22,23]. From this perspective, linguistic structures are not arbitrary but are motivated by the way people perceive and structure the world.

A cornerstone of this framework is Conceptual Metaphor Theory (CMT), articulated by Lakoff and Johnson [24]. They demonstrated that metaphor is not merely a decorative linguistic device but a fundamental cognitive mechanism through which people understand one conceptual domain (the target domain) in terms of another (the source domain). For example, the research systematically conceptualizes ARGUMENT (target) in terms of WAR (source), leading to expressions like *attacking a weak point* or *defending a position*. This study posits that the divergent ways in which scholars use GNs may reflect a reliance on different underlying conceptual metaphors for the abstract domain of academic research. For instance, a preference for collocations that mark time, such as *previous study* and *current study*, suggests the activation of the conceptual metaphor ACADEMIC PROGRESS IS A JOURNEY, where research is conceptualized as a linear progression. Conversely, a preference for objectifying collocations like *the case* and *the argument* points to a more spatialized metaphor, such as THE STUDY IS A KNOWLEDGE CONTAINER, where knowledge is an object to be encapsulated and examined. This use of a CONTAINER image schema is a specific way to structure a broader conceptual SPACE, where ideas are treated as objects that can be positioned relative to one another. These underlying metaphors are built from fundamental cognitive structures known as image schemas (e.g., CONTAINER, PATH, SOURCE-PATH-GOAL), which are pre-conceptual structures arising from our bodily interactions with the world [25].

### 2.4 L1 rhetorical and grammatical influence

While cognitive linguistics offers one powerful explanatory lens, it is crucial to consider alternative or complementary frameworks, particularly in the context of second language writing. A substantial body of research in contrastive rhetoric and L2 writing points to the significant influence of L1 rhetorical traditions and grammatical structures on L2 production. The patterns observed in this study could, therefore, be interpreted not only as evidence of different cognitive models but also as manifestations of L1 transfer.

Rhetorically, some scholars have argued that traditional Chinese argumentation may favor different modes of development and evidence compared to the linear, thesis-driven model often promoted in Anglophone academic contexts. While this is a complex and debated area, it is plausible that certain preferences for framing research—for instance, as a chronological narrative of discovery—may be influenced by established L1 rhetorical conventions that L2 writers bring to their English writing tasks. This perspective aligns with an academic literacies approach, which views writing practices as socially situated and culturally shaped [26].

Grammatically, the influence of L1 is even more direct and well-documented. One of the most significant points of divergence between Chinese and English is the absence of an article system in Chinese. Chinese-speaking learners of English consistently demonstrate difficulty with the correct and idiomatic use of definite (*the*) and indefinite (*a/an*) articles [7]. This grammatical difference provides a direct, alternative explanation for some of the key collocational patterns under investigation. For example, a lower frequency of the collocation *the study* in the Chinese expert corpus could be interpreted not only as a cognitive preference for a different framing device but also as a residual effect of L1 grammatical transfer, where the use of the definite article is less automatic or conventional even among highly proficient writers. An effective analysis must therefore hold these two potential explanations—cognitive-rhetorical preference and L1 grammatical transfer—in productive tension.

### 2.5 A cognitively-informed, rhetorically-aware approach to L2 writing pedagogy

The integration of these theoretical perspectives—local grammar, cognitive linguistics, and L1 transfer—carries profound implications for L2 writing pedagogy. A cognitive-linguistic perspective suggests that to effectively address the deep-seated difficulties faced by students, instruction must engage with the underlying cognitive habits that motivate their linguistic choices. Research has shown that making learners explicitly aware of conceptual metaphors can significantly improve their comprehension and production of figurative language [9,27]. This study seeks to extend that pedagogical principle to the more subtle, conventionalized metaphors embedded in academic writing.

By first diagnosing the specific metaphorical systems that may be preferentially activated in different academic discourse traditions (via a corpus analysis of GN collocations), the research can then develop pedagogical materials designed to make these invisible cognitive frameworks visible to L2 writers [28,29]. However, this approach must be complemented by an explicit focus on areas of known L1 influence. The objective is to shift the instructional focus from merely correcting surface-level errors to fostering a deeper “metaphorical awareness” [30] and rhetorical flexibility. This involves helping students understand that mastering academic English requires not just learning new words and syntactic structures, but also learning to recognize and skillfully navigate the different ways of thinking and arguing that are encoded in the language, while simultaneously addressing specific grammatical challenges stemming from their L1 [31]. This integrated approach aligns with a growing movement advocating for the use of corpus linguistics (Data-Driven Learning, or DDL) and cognitive linguistics to create more effective, evidence-based, and cognitively-informed language teaching practices [32,33].

## 3. Method

### 3.1 Research design and corpora

This study employs a corpus-based, comparative research design to investigate the usage patterns of GNs in academic writing. This approach uses corpus data to empirically test hypotheses derived from linguistic theory and classroom observation, revealing the relationship between frequency of occurrence and the representativeness of specific linguistic phenomena [34]. The ultimate aim is to use these findings to inform targeted pedagogical interventions. To this end, two specialized corpora were purpose-built for this study:

**Chinese Academic Written English Corpus (CAWEC):** This corpus serves as the target corpus, intended to reflect the writing patterns of expert Chinese scholars writing in English. It contains 134 research articles, totaling 982,976 tokens.
**Inner-Circle Affiliated Written English Corpus (ICAWEC):** This corpus serves as the reference or baseline corpus. It contains 150 research articles, totaling 1,041,886 tokens (see Table 1).

**Table 1:** Corpus information.

Corpus	Number of papers	Token count
CAWEC	134	982,976
ICAWEC	150	1,041,886

All texts for both corpora were selected based on the following criteria:

**Source Journals:** The articles were sourced from four prominent, high-impact journals in the field of applied linguistics: *Applied Linguistics*, *Journal of English for Academic Purposes*, *Journal of Second Language Writing*, and *English for Specific Purposes*. This ensures the data is authoritative and representative of the target genre.
**Publication Period:** All articles were published between 2005 and 2024. This time frame was chosen to ensure the language reflects contemporary academic writing conventions.
**Author Background:** For the CAWEC, “expert” status was operationally defined by successful publication in these high-impact international journals. To identify Chinese scholars, we filtered for articles where the first author was affiliated with a mainland Chinese institution. We further verified this by reviewing author biographical notes to exclude international scholars visiting Chinese institutions, ensuring the corpus reflects the rhetorical habits of scholars from a Chinese cultural and linguistic background who have achieved international publication success. For the ICAWEC, articles were authored by scholars affiliated with inner-circle English-speaking countries (e.g., USA, UK, Australia, Canada).

It is important to acknowledge the limitations of this author identification method. Institutional affiliation is not a definitive proxy for a writer’s L1 status, and the ICAWEC likely contains articles by highly proficient L2 English users. Therefore, this study is not positioned as a simplistic native vs. non-native comparison. Rather, it is a comparison between the published writing of scholars affiliated with Chinese institutions and those affiliated with “inner-circle” institutions, treating the latter corpus as a data-driven proxy for the high-frequency conventions found in the target publication venues. To mitigate the potential confounding effects of sub-disciplinary topic and research methodology, articles were sampled proportionally from a range of topics within applied linguistics (e.g., SLA, discourse analysis, language testing) and included a balanced mix of qualitative, quantitative, and mixed-methods studies in both corpora.

### 3.2 Instruments used

The analysis was conducted using two primary software tools:

**Sketch Engine:** This corpus analysis tool was used for building the corpora, extracting keywords and collocations, and retrieving concordance lines for qualitative analysis.
**SPSS, Version 26.0:** This software was used for all statistical analyses. The Chi-square (χ^2^) test was employed to determine whether the observed frequency differences in the use of GNs and their collocations between the two corpora were statistically significant. All frequency data and statistical calculations were subjected to a complete manual audit to ensure accuracy.

### 3.3 Research procedures

The research was carried out following a systematic five-step procedure:

**Step 1: High-Frequency General Noun Identification.** Based on previous research [5,6] and an initial frequency analysis, a preliminary list of potential GNs was compiled. A comparative frequency analysis was then performed. Chi-square tests were conducted on the normalized frequencies (per million words) to account for the slight difference in corpus size and identify nouns whose frequency of use differed significantly between CAWEC and ICAWEC. This process yielded a final list of 27 high-frequency GNs where the usage difference was statistically significant (p < 0.05).
**Step 2: Semantic Group Classification.** Drawing on functional classifications in previous studies [35,36], the 27 identified GNs were categorized into six semantic groups: Research, Result, Discussion, Example, Context, and Method. This classification framework was selected because it moves beyond simple grammatical categorization to capture the *epistemological function* of the noun. By grouping nouns into categories like “Research” (process-oriented) versus “Result” or “Discussion” (product-oriented), we can quantitatively assess whether different writer groups prioritize different stages of knowledge construction. A Chi-square analysis was then performed on the cumulative frequencies of these groups.
**Step 3: Collocation Pattern Analysis.** Using Sketch Engine, the research identified the most frequent and statistically significant L1 collocations (the first word to the immediate left of the target GN). A Chi-square test was then run on these collocation pairs (e.g., *current study*, *the study*) to pinpoint significant differences in preference between the two corpora [37].
**Step 4: Local Function and Context Analysis.** This step involved a qualitative analysis to interpret the quantitative findings. For each of the most statistically significant collocational pairs, a random sample of 100 concordance lines from each corpus (for a total of 200 lines per pair) was extracted for qualitative analysis of local discourse functions (e.g., temporal positioning, objectification). The research systematically categorized these functions to build a picture of the divergent rhetorical strategies [38].
**Step 5: Cognitive and Rhetorical Motivation Analysis.** In the final step, the research synthesized the findings through the theoretical lenses of cognitive linguistics and L1 transfer. The research analyzed how the observed patterns of GN collocation and function might reflect underlying conceptual metaphors (e.g., “Journey” vs. “Container”) and how they might also be explained by L1 rhetorical and grammatical influences. This allowed us to move from a description of linguistic differences to an explanation of the potential motivations behind them [39].

## 4. Results and discussion

### 4.1 Overall distribution of high-frequency general nouns

The initial comparative analysis identified 27 GNs whose frequencies of use differed to a statistically significant degree (p < 0.05). Table 2 provides a comprehensive overview of these differences, presenting the raw frequency, the corrected normalized frequency per million words, and the Chi-square test results for each noun.

**Table 2:** Distribution of high-frequency general nouns and Chi-square test results.

Items	CAWEC Freq.	ICAWEC Freq.	CAWEC Norm. Freq.	ICAWEC Norm. Freq.	Dir.	Chi-square	Sig.
**research**	5094	4319	5182	4145	+	117.27	0.0000
**study**	3113	3025	3167	2903	+	11.53	0.0007
**studies**	1868	1451	1900	1393	+	79.34	0.0000
**findings**	1290	869	1312	834	+	108.17	0.0000
**questions**	1153	744	1173	714	+	113.29	0.0000
**results**	1124	1036	1143	994	+	10.41	0.0013
**time**	906	1175	922	1128	–	20.72	0.0000
**differences**	900	664	916	637	+	50.38	0.0000
**process**	770	687	783	659	+	10.63	0.0011
**strategies**	755	682	768	655	+	9.03	0.0027
**functions**	658	448	669	430	+	52.66	0.0000
**discussion**	648	548	659	526	+	14.99	0.0001
**approach**	641	773	652	742	–	5.72	0.0168
**model**	537	387	546	371	+	33.52	0.0000
**role**	536	477	545	458	+	7.56	0.006
**ability**	484	330	492	317	+	38.39	0.0000
**effect**	440	373	448	358	+	9.90	0.0017
**sections**	438	387	446	371	+	6.64	0.0099
**argument**	430	217	437	208	+	82.44	0.0000
**question**	416	547	423	525	–	10.82	0.001
**examples**	405	521	412	500	–	8.39	0.0038
**case**	402	629	409	604	–	37.32	0.0000
**difference**	400	283	407	272	+	27.06	0.0000
**ways**	327	518	333	497	–	32.43	0.0000
**form**	264	423	269	406	–	27.76	0.0000
**need**	262	460	267	442	–	42.96	0.0000
**fact**	255	430	259	413	–	34.70	0.0000

The data reveal a stark divergence in lexical preferences. The CAWEC corpus shows a significantly higher normalized frequency for GNs that explicitly label core components of the research process. Most notably, the nouns *research* (χ^2^ = 117.27, p < 0.0001), *studies* (χ^2^ = 79.34, p < 0.0001), *findings* (χ^2^ = 108.17, p < 0.0001), and *questions* (χ^2^ = 113.29, p < 0.0001) are all heavily favored by writers in the CAWEC. This strong preference suggests a discourse style that prioritizes explicit metadiscursive signposting, clearly demarcating the different stages and elements of the research narrative.

Conversely, the ICAWEC corpus demonstrates a significantly stronger preference for a different set of GNs, primarily those related to argumentation, conceptualization, and exemplification. Nouns such as *case* (χ^2^ = 37.32, p < 0.0001), *fact* (χ^2^ = 34.70, p < 0.0001), *need* (χ^2^ = 42.96, p < 0.0001), and *question* (singular, χ^2^ = 10.82, p < 0.001) are all used significantly more often by writers in the ICAWEC. This pattern points toward a rhetorical style that may place greater emphasis on the construction of a conceptual argument, the evaluation of evidence, and the grounding of claims in specific, illustrative instances.

### 4.2 Semantic group analysis

To better understand the broader trends underlying these individual lexical preferences, the 27 GNs were classified into six semantic groups. Table 3 presents the results of the Chi-square tests on the cumulative frequencies for each group.

**Table 3:** Results of Chi-square tests on semantic group frequencies.

Semantic Groups	CAWEC Freq.	ICAWEC Freq.	CAWEC Norm. Freq.	ICAWEC Norm. Freq.	Dir.	Chi-square	Sig.
**Research**	11365	9664	11562	9275	+	257.03	0.0000
**Result**	2864	2365	2914	2270	+	81.11	0.0000
**Discussion**	2647	2056	2693	1973	+	112.68	0.0000
**Example**	1062	1580	1080	1516	–	73.48	0.0000
**Context**	2422	2652	2464	2545	–	1.31	0.2524
**Method**	2260	2360	2299	2265	+	0.24	0.622

The statistical analysis of the semantic groups powerfully confirms and clarifies the patterns observed at the individual noun level. Four of the six analyzed groups show highly significant differences:

**Research group:** This group, comprising nouns that label the research endeavor itself (e.g., *research, study*), is used with overwhelmingly higher frequency in the CAWEC (χ^2^ = 257.03, p < 0.0001). This reinforces the observation that the writing in this corpus tends to employ a high degree of explicit meta-discursive framing.
**Result group:** Writers in the CAWEC also demonstrate a significantly higher preference for this group of nouns (e.g., *results, differences*) (χ^2^ = 81.11, p < 0.0001), underscoring a rhetorical focus on the clear and explicit presentation of research outcomes.
**Discussion group:** This group, containing nouns central to argumentation and inquiry (e.g., *discussion, questions, argument*), is also used more frequently in CAWEC (χ^2^ = 112.68, p < 0.0001).
**Example group:** The trend reverses dramatically for this group. Writers in the ICAWEC show a much stronger preference for nouns used in exemplification (e.g., *case, examples, fact*) (χ^2^ = 73.48, p < 0.0001). This is a key finding, suggesting a greater reliance in the Anglophone-affiliated writing on grounding abstract claims in concrete, specific evidence presented as cases or facts.

Notably, the Context and Method groups showed no statistically significant difference. This suggests that in describing the background and methodological framework of their research, both groups of writers adhere to broadly similar rhetorical conventions.

### 4.3 Analysis of collocation patterns and local functions

The research turns to a micro-level analysis of the L1 collocational patterns for the GNs within the four key semantic groups.

#### 4.3.1 The “Research” Group: Temporal profiling versus objectification.

The collocational patterns for GNs like *study* and *research* expose a divergence in how the research enterprise is framed. Writers in the CAWEC heavily favor collocations that perform a function of temporal profiling and subjective ownership. The use of temporal markers is striking, with “current study” (χ^2^ = 51.17), “present study” (χ^2^ = 17.50), and “previous studies” (χ^2^ = 103.18) all being used with significantly higher frequency. This pattern explicitly positions the research on a chronological timeline. Concurrently, these writers show a significant preference for ownership markers like “our study” (χ^2^ = 15.99) and “our research” (χ^2^ = 6.46), which foregrounds the researchers’ active role, as shown in Table 4.

**Table 4:** Collocation distribution of research-group general nouns.

Collocates	Items	CAWEC Freq.	ICAWEC Freq.	CAWEC Norm. Freq.	ICAWEC Norm. Freq.	Dir.	Chi-square	Sig.
**current**	study	211	94	215	90	+	51.17	0.0000
**this**	study	1088	910	1107	873	+	27.72	0.0000
**present**	study	325	241	331	231	+	17.50	0.0000
**our**	study	240	170	244	163	+	15.99	0.0001
**the**	study	384	560	391	537	–	23.09	0.0000
**previous**	studies	279	93	284	89	+	103.18	0.0000
**the**	research	607	471	618	452	+	25.71	0.0000
**published**	research	59	17	60	16	+	24.59	0.0000
**previous**	research	236	156	240	150	+	20.87	0.0000
**our**	research	59	36	60	35	+	6.46	0.0110
**this**	research	56	121	57	116	–	19.59	0.0000
**our**	findings	115	65	117	62	+	16.36	0.0001

In contrast, a more nuanced pattern emerges in the use of the definite article *the*. While writers in the ICAWEC show a significant preference for objectifying the research act as “the study” (χ^2^ = 23.09), writers in the CAWEC show an even stronger preference for the collocation “the research” (χ^2^ = 25.71). The local function of this pattern is one of objectification. It frames the research not as a personal or time-bound activity but as a discrete, self-contained object that can be referenced as a point of shared knowledge [40]. This suggests that both groups use the definite article to create shared reference points, but they tend to objectify different aspects of the research process—the ICAWEC writers focusing on the specific ‘study’ as an entity, and the CAWEC writers focusing on the broader ‘research’ field or process.

#### 4.3.2 The “Discussion” Group: Systematic framing versus focused inquiry.

The framing of questions and arguments also reveals divergent strategies. CAWEC writers overwhelmingly prefer the plural form “research questions” (χ^2^ = 159.60). Qualitative analysis of concordance lines confirms these are frequently presented as a systematic list, performing a local function of systematic construction by laying out the entire framework of inquiry. They also favor the formal label “the discussion” (χ^2^ = 33.75), treating it as a clearly demarcated section of the paper. Conversely, ICAWEC writers show a significant preference for the singular forms “the question” (χ^2^ = 10.53) and “the argument” (χ^2^ = 20.93). This suggests a more focused, critique-driven approach, where the discourse is structured around a single, core “question” under debate, as shown in Table 5.

**Table 5:** Collocation distribution of discussion-group general nouns.

Collocates	Items	CAWEC Freq.	ICAWEC Freq.	CAWEC Norm. Freq.	ICAWEC Norm. Freq.	Dir.	Chi-square	Sig.
**the**	argument	35	91	36	87	–	20.93	0.0000
**the**	discussion	141	63	143	60	+	33.75	0.0000
**the**	question	63	112	64	107	–	10.53	0.0012
**research**	questions	455	159	463	153	+	159.60	0.0000

#### 4.3.3 The “Result” Group: Quantitative assertion versus intertextual linking.

When presenting outcomes, CAWEC writers exhibit a strong preference for quantitative assertion. The collocations “significant difference” (χ^2^ = 48.71) and “significant differences” (χ^2^ = 19.25) are defining features of this corpus. The local function is to explicitly assert the empirical certainty of the findings. They also favor definite referents like *the difference(s)*, which serve to frame the results as discrete packages of information. In contrast, ICAWEC writers show a statistically significant preference for the demonstrative “these results” (χ^2^ = 4.04). The function of this pattern is one of intertextual connection. It dynamically links the results just presented back into the ongoing stream of argument, weaving them into the discourse rather than presenting them as standalone facts, as shown in Table 6.

**Table 6:** Collocation distribution of result-group general nouns.

Collocates	Items	CAWEC Freq.	ICAWEC Freq.	CAWEC Norm. Freq.	ICAWEC Norm. Freq.	Dir.	Chi-square	Sig.
**significant**	difference	111	31	113	30	+	48.71	0.0000
**the**	difference	89	66	91	63	+	4.53	0.0331
**significant**	differences	119	64	121	61	+	19.25	0.0000
**the**	differences	131	78	133	75	+	16.16	0.0001
**these**	results	38	62	39	60	–	4.04	0.0444

#### 4.3.4 The “Example” Group: The power of shared reference.

As established by the semantic group analysis, ICAWEC writers use Example-group nouns more frequently overall. The collocation data in Table 7 reveals that this preference is driven almost entirely by the definite article *the*. The collocations “the case” (χ^2^ = 33.50$), “the fact” (χ^2^ = 23.97$), and “the examples” (χ^2^ = 12.18$) are all significantly preferred by writers in the ICAWEC. The local function of this pattern is to frame the instance not merely as an illustration, but as a piece of shared, recognized evidence. The use of *the case* or *the fact* presupposes that this is an entity the reader is, or should be, familiar with, thereby establishing it as a definitive cognitive reference point for the argument that follows [41]. This rhetorical strategy of spatializing evidence—transforming an example into a landmark within the argumentative landscape—is a quintessential feature of the conceptual model discussed next.

**Table 7:** Collocation distribution of example-group general nouns.

Collocates	Items	CAWEC Freq.	ICAWEC Freq.	CAWEC Norm. Freq.	ICAWEC Norm. Freq.	Dir.	Chi-square	Sig.
**the**	case	128	253	130	243	–	33.50	0.0000
**the**	examples	14	43	14	41	–	12.18	0.0005
**the**	fact	114	213	116	204	–	23.97	0.0000

## 5. Discussion

### 5.1 From dichotomy to divergent rhetorical tendencies for L2 writers

The multitude of statistical differences, when viewed holistically, can be interpreted through two distinct rhetorical strategies, which are presented as heuristic frameworks rather than absolute cognitive realities. These frameworks are particularly useful for diagnosing the challenges L2 writers face when navigating the conventions of Anglophone academia.

#### 5.1.1 The “Journey” Heuristic: Temporal, linear, and researcher-centric.

The patterns dominant in the CAWEC corpus are consistent with the conceptual metaphor ACADEMIC PROGRESS IS A JOURNEY. This model frames the act of research and writing as a chronological, linear process led by the researcher. This rhetorical stance is realized through several key linguistic features:

**Temporal Profiling:** The significant preference for temporal markers like *current study* and *previous findings* explicitly activates a timeline image schema. The research is situated on a historical continuum, creating a narrative of progression over time [42]. For an L2 writer, this can feel like a safe and logical way to structure a paper, recounting the research process as it happened.
**Path Schema Activation:** Phrases common in this writing style activate the SOURCE-PATH-GOAL image schema [25], where a research “gap” is the starting point (SOURCE), the investigation is the movement along the PATH, and the findings are the destination (GOAL). This provides a clear, narrative-like structure.
**Researcher Subjectivity:** The heavy use of ownership markers like “our study” makes the researchers the explicit agents of this journey. While this foregrounds the human effort behind the research process [43], for L2 writers it can also reflect a less confident stance, emphasizing their personal involvement rather than the objective authority of the findings themselves.
**Systematic Construction:** The preference for laying out multiple research questions in a list format functions as a roadmap provided at the beginning of the journey, enhancing the sense of a structured, linear progression.
**Asserting the Destination:** The frequent use of *significant difference* and *the results show* serves to announce the arrival at the journey’s destination with empirical certainty.

In this model, coherence is achieved through temporal sequencing and explicit signposting. However, this interpretation must be considered alongside the potential influence of L1 rhetorical traditions, which may also favor a narrative or chronological unfolding of ideas and may be transferred into L2 academic writing.

#### 5.1.2 The “Container/Space” Heuristic: Spatial, networked, and argument-centric.

In contrast, the patterns dominant in the ICAWEC are consistent with a different set of metaphors, primarily THE STUDY IS A KNOWLEDGE CONTAINER and ACADEMIC DISCOURSE IS A CONCEPTUAL SPACE. This model frames writing as the construction of a conceptual space where knowledge-objects are defined and related to one another. This approach often represents a significant rhetorical hurdle for L2 writers.

**Objectification and Encapsulation:** The preference for *the study* objectifies the research into a discrete entity, activating the CONTAINER image schema [23]. The study is a “vessel” that holds contents, a strategy that backgrounds the researcher’s role and creates an impersonal, authoritative stance that many L2 writers find difficult to adopt.

**Spatial Landmarks and Positioning:** The preference for *the case, the fact,* and *the argument* serves to populate the conceptual space with established landmarks [22]. The argument is built by positioning the current study’s claims in relation to these fixed points, a sophisticated rhetorical move requiring considerable confidence.

**Critique-Driven Dialogue:** The preference for the singular *the question* over a list of questions frames the discourse as a focused inquiry into a central problem, exploring the relationships radiating from a central node [44].

**Intertextual Weaving:** The use of demonstratives like *these results* functions to weave findings directly back into the argumentative fabric, treating them as nodes in a larger conceptual network [45].

This rhetorical tendency may be particularly challenging for L2 writers whose L1 does not have an equivalent article system. The difficulty in mastering the nuances of the definite article could contribute to a lower frequency of these objectifying patterns, independent of any underlying cognitive preference. Therefore, the observed differences are likely the result of a complex interplay between preferred rhetorical strategies and the concrete effects of L1 grammatical transfer, presenting a significant area of need for targeted L2 writing instruction.

### 5.2 From diagnosis to pedagogy: Fostering rhetorical flexibility in the L2 writing classroom

The diagnostic findings from this study provide a clear mandate for a more nuanced L2 writing pedagogy. Instruction must evolve beyond a focus on form to address the underlying rhetorical frameworks that govern effective academic writing, while also tackling specific L1-related challenges. The goal is not to eradicate the “Journey” model but to make L2 writers “rhetorically bilingual,” able to consciously select from a wider repertoire of strategies to achieve their communicative goals. The study proposes a four-pronged pedagogical framework based on this principle.

#### Cultivating Metacognitive Awareness of Rhetorical Models.

The foundational step is to make the implicit “Journey” and “Space” heuristics explicit to L2 writers.

*Instructional Goal:* Students should be **able** to identify and articulate the core rhetorical strategies and resulting authorial stances at play in an academic text.

*Sample Activity:* Argument Structure Mapping: Students **are** given two short, parallel text excerpts, one exemplifying a “Journey” structure and one a “Space” structure. In groups, they first highlight all GNs and their key collocates. They then create a visual map of each argument. For the “Journey” text, this might be a linear timeline showing *previous research* - > *our research questions* - > *the current study* - > *our findings*. For the “Space” text, it might be a concept map with the central problem at the center, linked to nodes like “the evidence, the case of X, and the argument that...” This contrastive analysis makes the abstract rhetorical concepts tangible and debatable.

#### Data-Driven Learning (DDL) for Pattern Recognition.

Leverage corpus tools to allow students to discover the patterns for themselves, fostering learner autonomy.

*Instructional Goal:* Students should use **concordance** data to deduce the different rhetorical functions and stances associated with key collocational patterns.

*Sample Activity:* Concordance-Based Stance Analysis: The **instructor** provides students with curated concordance lines for the noun *study*. One set shows lines for “our study”, another for “this study”, and a third for “the study”. Students work in pairs to analyze the lines and answer guiding questions: “In which set does the author sound most personally involved? Most objective? In which set is the author simply pointing to a part of their own text? What effect does this choice have on you as a reader?” Through this inductive process, students discover that the choice of determiner is not random but a powerful tool for modulating authorial stance and voice [30].

#### Focusing on Functional Repertoires through Rhetorical Rewriting.

The aim is not to replace one model with another, but to expand students’ rhetorical repertoires.

*Instructional Goal:* Students should be able to select GN collocations strategically to achieve a desired rhetorical effect, such as projecting more authority or creating a stronger narrative flow.

*Sample Activity:* Rhetorical Transformation: Students are given a short paragraph from their own writing that predominantly uses a “Journey” style (e.g., “In our study, we first looked at previous research. Then, our research questions were... Our findings showed a significant difference.”). Their task is to rewrite the paragraph to adopt a “Space” model stance (e.g., “The study is situated within a body of literature that has...” “The central question is whether...” “Evidence from the data suggests a significant difference, a finding that supports the argument that...”). A follow-up peer review and discussion focuses on the changes in tone, authority, and focus, teaching flexibility and demonstrating that different models are suited to different rhetorical purposes.

#### Addressing L1 Influence Directly through Targeted Practice.

Since the “Space” model relies heavily on lexico-grammatical features that are challenging for many L2 writers, instruction must address these directly and explicitly.

*Instructional Goal:* Students should gain explicit control over the use of articles with GNs to achieve specific discourse functions like objectification and establishing shared knowledge.

*Sample Activity:* Article Function Drills: Provide students with cloze exercises or sentence-completion tasks focusing specifically on GNs in authentic academic contexts. For example: “____ previous study by Smith (2018) found X. However, ____ study presented here uses a different methodology. The goal of ____ research is to...” Students must fill in the blanks with *a*, *the*, or zero article, and then justify their choices based on discourse function (e.g., introducing a new entity, referring to a specific, shared entity, referring to a general field). This directly connects the pedagogical intervention to the L1 transfer issue raised in the analysis.

By implementing this cognitively-informed and rhetorically-aware pedagogical framework, the research can move L2 students beyond the frustrating cycle of surface-error correction and empower them with the conceptual tools needed to navigate the complex rhetorical landscape of international academic communication [46].

## 6. Conclusion

This study was motivated by a direct pedagogical need: to understand and address the persistent difficulties L2 writers face with the nuanced use of general nouns in academic writing. Through a large-scale, comparative corpus analysis, this research has moved beyond a simple description of linguistic features to propose a multi-causal model of cross-cultural rhetorical differences that has significant implications for L2 writing instruction.

The major findings of this study are threefold. First, the research has demonstrated with robust statistical evidence that writers in the CAWEC and ICAWEC exhibit systematically different preferences in their use of GNs and their collocations. Writers in the CAWEC favor nouns and patterns that explicitly frame the research process and its results, while writers in the ICAWEC prefer those that build conceptual arguments and ground them in evidence. Second, the research has argued that these linguistic patterns can be interpreted through two distinct, underlying heuristic models: a temporal, linear “Journey” model and a spatial, networked “Container/Space” model. Third, the research has posited that these divergent tendencies are likely the product of a complex interplay between cognitive-rhetorical preferences and the direct influence of L1 grammatical and rhetorical transfer, a key consideration for L2 writing theory.

The primary contribution of this research to the field of second language writing is the articulation of this “Journey” versus “Space” framework as both a diagnostic and pedagogical tool. It provides L2 writing educators with a powerful lens through which to understand student difficulties not as isolated errors, but as potentially logical consequences of applying a different rhetorical script. This understanding paves the way for a more effective, cognitively-informed pedagogy. The practical implications, detailed in the discussion, offer a clear framework for designing instructional materials and classroom activities that aim to cultivate metacognitive awareness, foster pattern recognition through data-driven learning, and expand students’ rhetorical repertoires.

However, this study is not without its limitations. Firstly, the corpora, while carefully constructed, were limited to the discipline of applied linguistics. The rhetorical patterns identified here may vary across different academic fields. Future research should therefore seek to validate these findings with multi-disciplinary corpora. Secondly, the method for identifying author background relied on institutional affiliation, which is an imperfect proxy for L1 status. Future studies could employ more robust methods, such as author surveys, to ensure more accurate corpus composition. Finally, the pedagogical interventions proposed in this paper are, at present, a theoretically-grounded framework. The logical next step for this line of inquiry is to undertake classroom-based action research to implement these teaching strategies and empirically measure their impact on L2 students’ writing quality, rhetorical flexibility, and cognitive awareness.

In conclusion, the seemingly minor choice between phrases like “our study” and “the study” can be a window into vastly different ways of constructing knowledge and authorial stance. For L2 writing educators, recognizing that students are often expertly navigating a “journey” in an academic world that increasingly values the ability to build a “space” is the crucial first step. By equipping them with the cognitive and linguistic maps needed to understand both terrains, it can help them move from being competent language learners to becoming confident and effective participants in the global academic conversation.

## Supporting information

S1 File Supplementary materials. This compressed file contains the complete dataset and analysis details underlying the study, organized into four folders: 1. **GN Reference Table:** Contains the initial lists of general nouns. 2. **Detailed Data of Each Semantic Groups Including Selection Process:** Documentation of the data selection steps and detailed statistics for each group. 3. **Data of Semantic Groups after Selection:** The final categorized datasets used for analysis.
(ZIP)
