# Supplementary figures and images for: Navigating the landscape of academic prose: A corpus-driven inquiry into rhetorical preferences and their pedagogical implications for advanced L2 writers
Source: PLoS One. 2026 Mar 5;21(3):e0343739. doi: 10.1371/journal.pone.0343739 (PMC12962540; doi:10.1371/journal.pone.0343739)

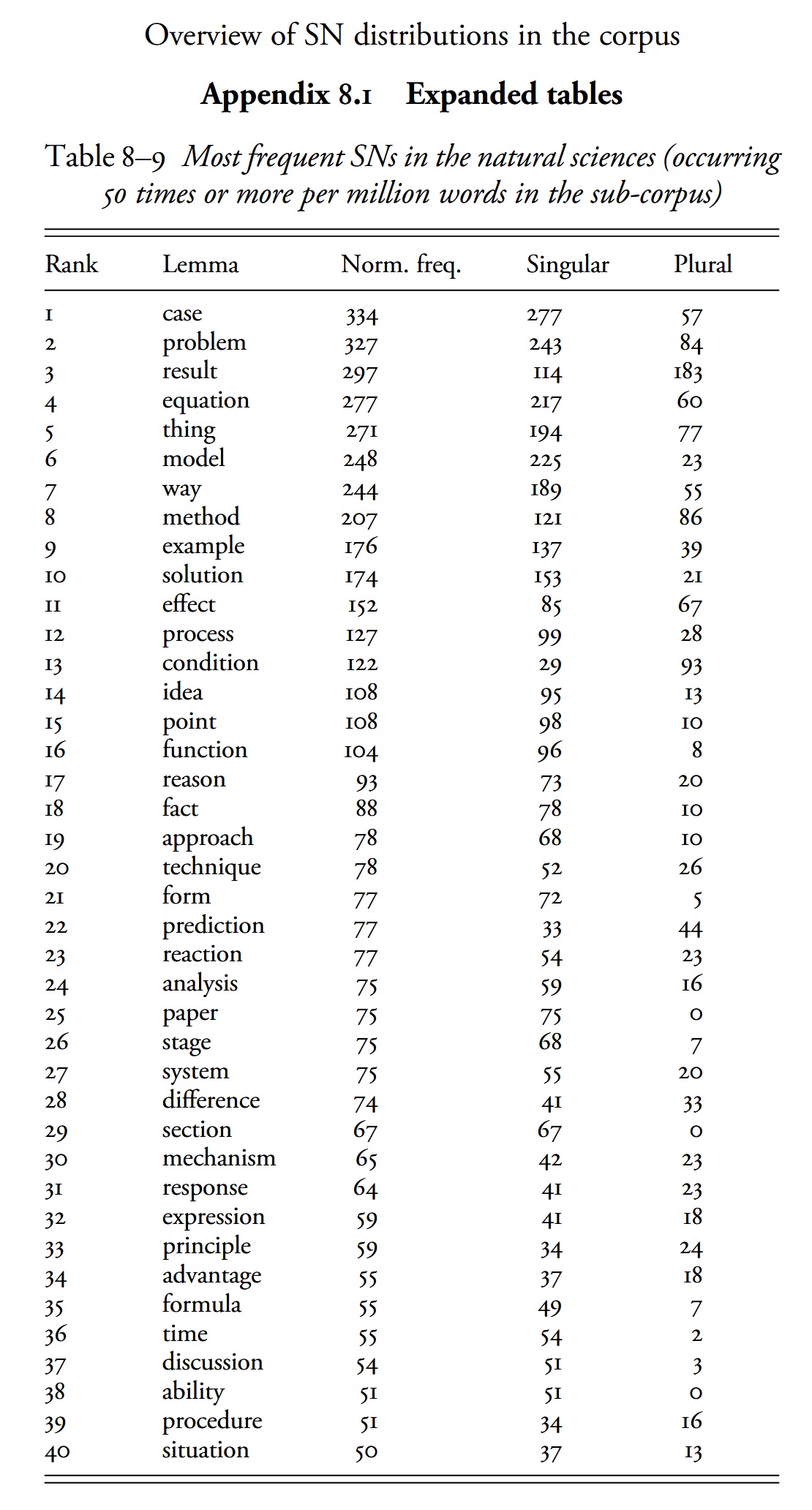

Supplement: S1 File — This compressed file contains the complete dataset and analysis details underlying the study, organized into four folders: 1. GN Reference Table: Contains the initial lists of general nouns. 2. Detailed Data of Each Semantic Groups Including Selection Process: Documentation of the data selection steps and detailed statistics for each group. 3. Data of Semantic Groups after Selection: The final categorized datasets used for analysis. (ZIP) [file pone.0343739.s001.zip › S1 File. supplementary materials/GN Reference Table/NS GN Reference Table.jpg]

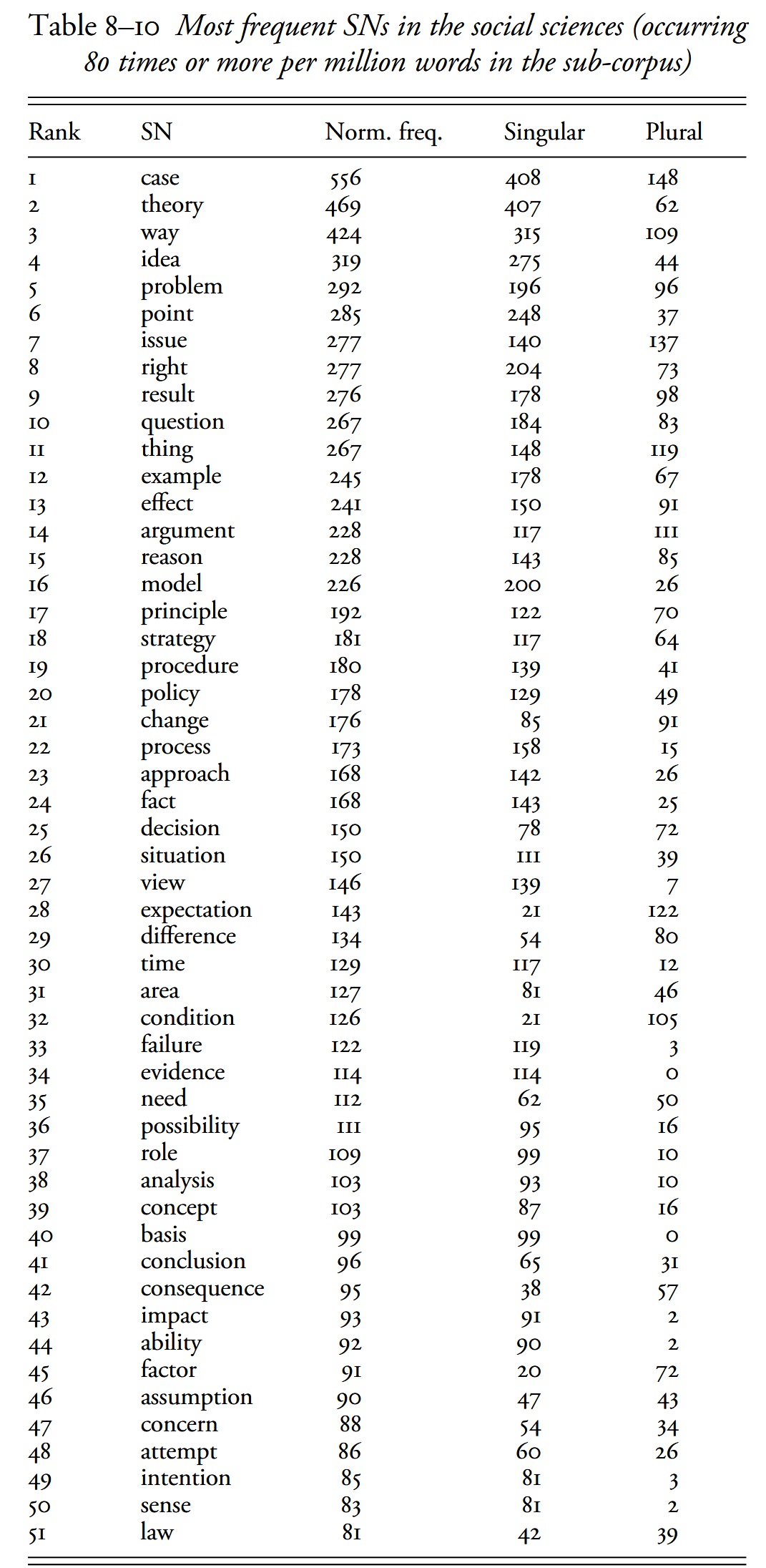

Supplement: S1 File — This compressed file contains the complete dataset and analysis details underlying the study, organized into four folders: 1. GN Reference Table: Contains the initial lists of general nouns. 2. Detailed Data of Each Semantic Groups Including Selection Process: Documentation of the data selection steps and detailed statistics for each group. 3. Data of Semantic Groups after Selection: The final categorized datasets used for analysis. (ZIP) [file pone.0343739.s001.zip › S1 File. supplementary materials/GN Reference Table/SS GN Reference Table.jpg]
